# Extreme Growth Increments Reveal Local and Regional Climatic Signals in Two *Pinus pinaster* Populations

**DOI:** 10.3389/fpls.2021.658777

**Published:** 2021-05-17

**Authors:** Joana Vieira, Cristina Nabais, Filipe Campelo

**Affiliations:** Centre for Functional Ecology, Department of Life Sciences, University of Coimbra, Coimbra, Portugal

**Keywords:** divergence, synchrony, extreme events, drought, global climate change

## Abstract

Tree rings are valuable proxies of past climate that allow inferring past growth responses to climate variability and extreme events, which is only possible considering that the relationship between tree growth and environmental conditions is linear and stable over time. However, in the last decades, divergent growth patterns have been observed in trees from the same forest stand, while unprecedented growth convergence was observed between trees from distant locations. Here, we use a new approach that considers convergent and divergent event years in two populations of *Pinus pinaster* Aiton in an altitudinal and oceanic-continental gradient to investigate what is triggering divergence and convergence in tree growth. The two study sites are Tocha (TCH), a plantation on sand dunes at low altitude near the ocean, and Serra da Estrela (SdE), a mountain plantation located at 1,100 m altitude, 100 km away from the ocean. The analysis of the climatic conditions in convergent growth years revealed that positive convergent growth was related to above average precipitation in previous winter and that negative convergent growth was related to below average precipitation during the growing season. Divergent growth revealed a temperature signal with warmer temperatures in spring promoting growth in SdE and growth reduction in TCH. Convergent growth was associated with a regional climatic signal, reinforcing the importance of precipitation in the Mediterranean region, and divergent growth to site conditions, revealing local adaptation. The information gathered in this study gives valuable insights on the response of *P. pinaster* to extreme climatic events, allowing for more adjusted management strategies of Mediterranean pine forests.

## Introduction

Dendroclimatology assumes that tree growth-climate responses are stable over time (Fritts, 1976). However, several studies have recently reported divergent growth trends and loss of sensitivity in the climatic response of trees (Jacoby and D’Arrigo, 1995; Briffa et al., 1998; Wilmking et al., 2020), the so-called “divergence problem” (D’Arrigo et al., 2008). The divergence problem is characterized by an offset between warmer instrumental temperatures and their underestimation in reconstruction models based on tree rings (D’Arrigo et al., 2008; Wilmking et al., 2020). It is defined as the weakening of temperature response in previous temperature-limited northern sites in the last decades and expressed as a loss in climate sensitivity or divergence in trend (D’Arrigo et al., 2008). For example, Wilmking et al. (2004) reported a divergence in trend in a study across Alaska, where some trees presented growth reduction while others increased growth in response to the recent temperature increase. The growth increase could be due to longer growing seasons, as a result of an earlier onset of tree growth in response to global warming (Rossi et al., 2011; Lugo et al., 2012). Trees that showed growth reduction revealed a shift in climatic sensitivity, with increasing temperature inducing drought stress, and the limiting factor shifting from temperature to precipitation (Wilmking et al., 2004, 2005).

Besides the divergence observed in temperature-limited sites, other studies have demonstrated an increased synchronization of tree growth at local and regional scales, which has been linked to climate change (Shestakova et al., 2016; Manzanedo et al., 2020). The concept of spatial synchrony in tree growth points to the convergence of changes in ring-width patterns among geographically distant populations (Liebhold et al., 2004; Shestakova et al., 2016). A global analysis of tree-ring growth over the past millennium has revealed that global synchrony in tree growth has increased since 1970, probably due to the recent warming caused by anthropogenic climate change (Manzanedo et al. 2020). Another example of increased forest growth synchrony at global scales was reported by Shestakova et al. (2016) that observed a synchrony between conifers growing in Spain and in Central Siberia, distant ∼1,000 km. The main climatic drivers of tree growth, and the climatic signal of tree rings, are probably changing and becoming more similar at wider spatial scales. This will have important ecological implications for forest growth and ultimately for the species geographic distribution (Bellard et al., 2012).

Most studies reporting increased growth synchrony or divergence as a result of climate change have been conducted in temperature-limited environments. However, how will trees respond in environments where climate-growth relationships are characterized by a complex interplay of temperature and precipitation signals (Liñán et al., 2012), such as the Mediterranean region? Previous dendrochronological studies in *Pinus pinaster* have demonstrated a strong positive relation of tree-ring width with previous winter and current spring precipitation, and a negative response to summer temperature (Vieira et al., 2009; Campelo et al., 2013; Nabais et al., 2014). Climate projections for the Mediterranean region predict an increase in the mean annual temperature from 3.3 to 4.1°C, a decrease of 11–17% of the total annual precipitation, and an increase of extreme events (Jacob et al., 2014). Dendrochronological studies can evaluate the effect of climate change-driven temperature increase and precipitation decrease; however, these studies are derived from correlative approaches that are not appropriate to detect the effect of extreme events, such as heatwaves, on tree growth. Analyzing extreme growth increments could be an efficient way to investigate the climatic conditions responsible for their formation. Event years are characterized by extreme growth (i.e., very narrow and very wide tree rings) observed at the individual tree level. On the other hand, a pointer year occurs when a significant proportion of trees presents the same event year, which indicates a significant change in the environmental conditions (Schweingruber, 1986). Pointer year and year-to-year correlative approaches are the two most-used methods to study the climatic signal in tree rings (Jetschke et al., 2019; Buras et al., 2020). Studies analyzing pointer years in the Mediterranean region, in Italy, found the formation of negative pointer years in drought years and positive pointer years in years with abundant summer precipitation (Battipaglia et al., 2009; Rita et al., 2014).

We have developed a new approach to compare convergent and divergent event years in two populations of *P. pinaster* growing at low and high altitudes. Our hypotheses are that (1) positive convergent years are associated with high precipitation in previous winter and spring; (2) negative convergent years are associated with drought years; and (3) divergent growth is driven by local climatic differences. We expect that the convergent and divergent event years reveal climatic signals that otherwise would not be detected by the traditional correlative approaches. Understanding the convergent and divergent growth of maritime pine forests growing at different altitudes, representing an oceanic-continental gradient, will provide insight into the response of this species to extreme climatic events, allowing for more adjusted management strategies of *P. pinaster* forests in the context of climate change.

## Materials and Methods

### Study Sites

**Figure 1:**
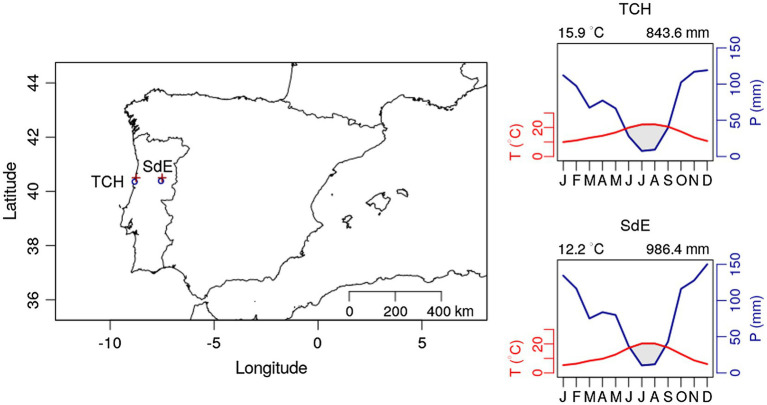
Geographic location of the two study sites Tocha (TCH) and Serra da Estrela (SdE; circle sign); and climatic diagram for TCH and SdE for the time period 1968–2008. Average temperature and total precipitation were downloaded for the nearest grid point from both study sites (plus sign “+”) at the Royal Netherlands Meteorological Institute Web site (see footnote 1).

The study sites were selected to represent the extreme altitudinal gradient of *P. pinaster* (maritime pine) plantations in continental Portugal (Figure 1). *Pinus pinaster* is the most representative conifer of the Portuguese forest representing 22% of the forest species (ICNF, 2019). In 10 years (2005–2015), 84.8 k hectares of the maritime pine forest were lost to wildfires in continental Portugal (ICNF, 2017), a direct consequence of climate change-induced drought intensity.

Both sites are even aged maritime pine plantations managed by the Portuguese forest services. Tocha (TCH) is located in the Perímetro florestal das dunas de Cantanhede (40°21'35''N; 8°49'10''W), a plantation on sand dunes at an altitude of 25 m above sea level. The trees were 45.3 ± 4.0 years old and presented a diameter at breast height of 38.7 ± 3.9 cm. Serra da Estrela (SdE) is located in Serra da Estrela National Park, a granite mountain range located in central Portugal with southwest-northeast orientation. Serra da Estrela is the tallest mountain in continental Portugal (1,993 m) and is divided into three altitudinal zones: below 800 m, from 800 to 1,600 m, and above 1,600 m. Our study site is located at 1,100 m, in the intermediate zone, on a mountain slope facing west (40°22'57''N, 7°33'11''W). The trees were 79.2 ± 12.6 years old at breast height and presented a diameter of 42.1 ± 8.4 cm. Both sites are located in central Portugal: TCH is close to the coastline and SdE is 100 km away from the Atlantic Ocean in a straight line, representing an oceanic-continental gradient from west to east (Figure 1).

**Figure 2:**
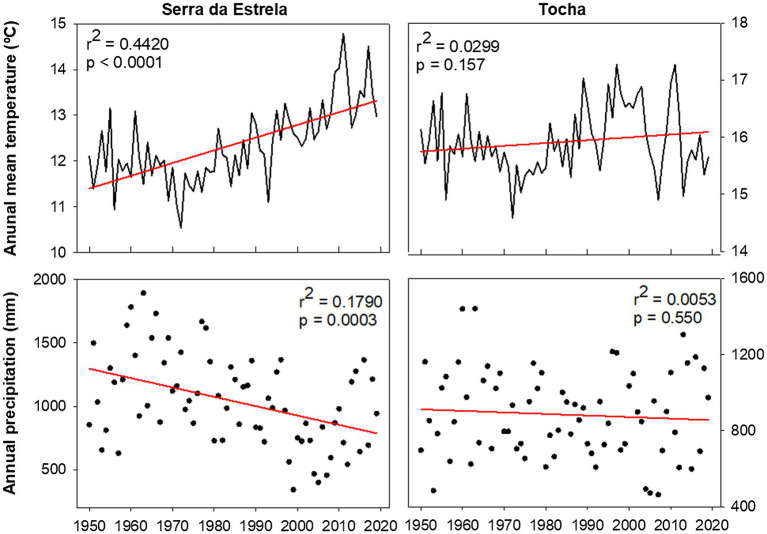
Average annual temperature and annual precipitation in SdE and TCH for the time interval 1950–2019 with the respective linear regression (red line).

Monthly climate data (maximum, mean, and minimum temperature and precipitation) and the Standardized Precipitation Evapotranspiration Index (SPEI; Vicente-Serrano et al., 2010) were extracted from the E-OBS-gridded climate data sets, E-OBS version 21.0e on a 0.25° regular grid.^1^ Climatic conditions were different between sites, reflecting their geographic location (Figure 1). Mean annual temperature for the 1968–2008 period was 14.9°C in TCH and 12.2°C in SdE (Figure 1). Minimum average temperatures in the winter months (December, January, and February) were 6.5°C in TCH and 2.3°C in SdE. Maximum average temperature in the summer months (June, July, and August) was 27.7 and 25.6°C in TCH and SdE, respectively. From 1968 to 2008, the increasing rate of mean annual temperature was higher in SdE than in TCH, as well as the decrease in total annual precipitation (Figure 2). The annual distribution of precipitation was similar between sites, with precipitation concentrated in the winter and spring months, decreasing significantly in the summer months (Figure 1). TCH registered a total annual precipitation of 844 mm and SdE 986 mm.

### Tree Selection, Sample Preparation, and Chronology Development

Thirty dominant trees were sampled in TCH in 2009 and 24 in SdE in 2011. Samples were taken at breast height using an increment borer. Two cores were taken from each tree from the north-south directions in TCH, whereas in SdE cores were taken perpendicular to the slope to avoid reaction wood. Cores were air-dried, mounted on a wooden support, and sanded with progressive finer sandpaper to highlight tree-ring limits. Tree rings were visually cross-dated using standard dendrochronological methods (Stokes and Smiley, 1996) and then measured to the nearest 0.01 mm using the R package *xRing* (Campelo et al., 2019).

For each site, chronologies for ring widths were developed by fitting a smoothing spline of 35-year to each tree-ring series. A first-order autoregressive model was applied to each ring-index series, and the resulting series were used to compute the residual chronologies. Chronologies were developed using the R packages dplR (Bunn, 2008) and detrendeR (Campelo et al., 2012). The quality of the chronologies was assessed by several dendrochronological statistics (Fritts, 1976) considering the common interval period (1968–2008; Table 1): mean sensitivity (MS), expressed population signal (EPS), first-order autocorrelation of raw data (Ar1), and mean correlation between trees (rbt). The coefficient of coherence was also quantified by average Gleichläufigkeit (Glk). EPS was calculated to determine the degree to which chronologies approach the hypothetically perfect chronology.

**Table 1:** Descriptive statistics of residual tree-ring chronologies.

	Tocha	Serra da Estrela
*Chronology length*	1953–2008	1910–2010
Number of trees	30	24
Number of cores	60	48
Mean tree-ring width	2.82	2.51
SD	1.30	1.13
Glk (%)	69.2	65.7
Mean sensitivity	0.30	0.26
Ar1	0.53	0.68
*Common interval (1968–2008)*		
Number of trees	30	24
Number of cores	58	48
rbt	0.38	0.34
EPS	0.96	0.95

SD, standard deviation; Glk (%), Gleichläufigkeit coefficient; Ar1, first-order autocorrelation; rbt, mean correlation between trees; EPS, expressed population signal.

The climatic signal of the tree-ring width residual chronologies was determined using Pearson correlation analysis between the tree-ring width indices and monthly minimum temperature, precipitation, and SPEI for the common period (1968–2008). Minimum temperature was selected because it represents a limiting factor for tree growth, especially in SdE. Regarding this, 3-months SPEI was selected to study the impact of short timescale droughts on tree growth, since droughts may act on growth at different timescales (Pasho et al., 2011).

### Event Year Analysis

Event years were identified using the relative growth change method (Meyer, 1998; Jetschke et al., 2019), by comparing each year radial increment to the average of the five previous years. Event year indices were calculated using tree-ring width chronologies. The 3 years with the highest (lowest) mean were defined as convergent positive (negative). Divergent years were identified by determining the difference between site chronologies. When the difference between site indices was >30% and the growth variation, >20% (when compared to the average of the five previous years), a divergent event year was identified. Event years were grouped in the following way: convergent positive, when both sites presented significant increase in growth (*t* = 2.593; *p* = 0.02), and convergent negative, when both sites presented significant decrease in growth (*t* = −3.566; *p* = 0.05). Divergent TCH+ years were characterized by growth increment in TCH and growth reduction in SdE, and divergent SdE+ years by the opposite.

Minimum temperature, precipitation, and SPEI of each group of event years were averaged and compared with the long-term mean (1968–2008) to determine the climatic conditions triggering them (Figures 5–7).

## Results

### Chronologies Characterization

**Figure 3:**
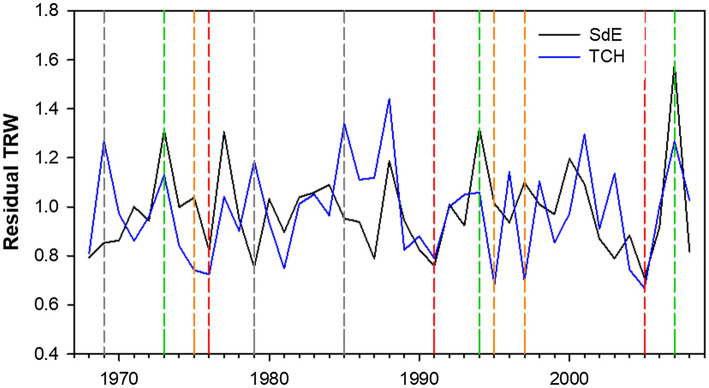
Residual tree-ring width (TRW) chronologies for SdE (black line) and TCH (blue line) for the common period 1968–2008. Vertical dash lines represent positive event years (convergent +; green line), negative event years (convergent −; red line), divergent years with TCH presenting a growth increment and SdE a growth reduction (Divergent TCH+; gray line), and the opposite (Divergent SdE+; orange line).

Tocha chronology covered 55 years (1953–2008), and SdE, 111 years (1910–2010; Figure 3; Table 1). The common interval between both sites (1968–2008) presented a high EPS (>0.9) and thus was used for further analysis (Table 1). The first-order autocorrelation was higher in SdE than in TCH. The mean tree-ring width in SdE was lower than in TCH, as well as the range of their ring widths 0.21–10.5 mm (SdE) vs. 0.26–12.9 mm (TCH).

### Climatic Signal

**Figure 4:**
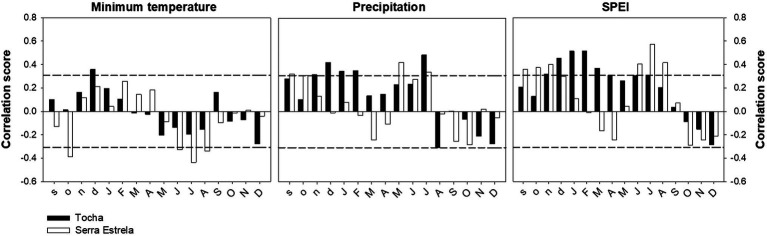
Correlations between TRW chronologies and minimum temperature, precipitation, and the Standardized Precipitation Evapotranspiration Index (SPEI) for TCH (black bars) and SdE (open bars) for the common interval 1968–2008. Horizontal dash lines represent significance level of *p* < 0.05.

The correlation analysis between tree-ring width chronologies and minimum temperature, precipitation, and SPEI revealed differences between sites (Figure 4). TCH presented a positive correlation with previous December minimum temperature. SdE presented negative correlations with the minimum temperature of previous October and from June to August (Figure 4). The precipitation signal was also different between sites with TCH presenting a positive correlation with previous November and December; current January, February, and July (Figure 4). TCH also presented a negative correlation with August precipitation. SdE responded positively to precipitation from the previous September and October; current May and July. Regarding SPEI, there was a positive correlation with previous September, October, and November and current June, July, and August in TCH, whereas in SdE there was a positive response from previous November to current July, except for May.

### Event Years

The event year analysis in both sites revealed that the convergent positive years were 2007, 1996, and 1973 and the convergent negative years were 2005, 1991, and 1976 (Table 2; Figure 3). The divergent growth years with growth increment in TCH and growth reduction in SdE (TCH+) were 1985, 1979, and 1969; the years presenting growth increment in SdE and growth reduction in TCH (SdE+) were 1997, 1995, and 1975 (Table 2; Figure 3).

**Table 2:** Event years for the SdE and TCH standard tree-ring width chronologies.

	Event year
Convergent +	2007
	1994
	1973
Convergent −	2005
	1991
	1976
Divergent TCH+	1985
	1979
	1969
Divergent SdE+	1997
	1995
	1975

Convergent years represent years where both chronologies presented a significant increment (Convergent +) or reduction (Convergent −) compared to the five previous years. Divergent TCH+ represent years where TCH presented a positive increment and SdE a negative one, and Divergent SE+ the opposite.

**Figure 5:**
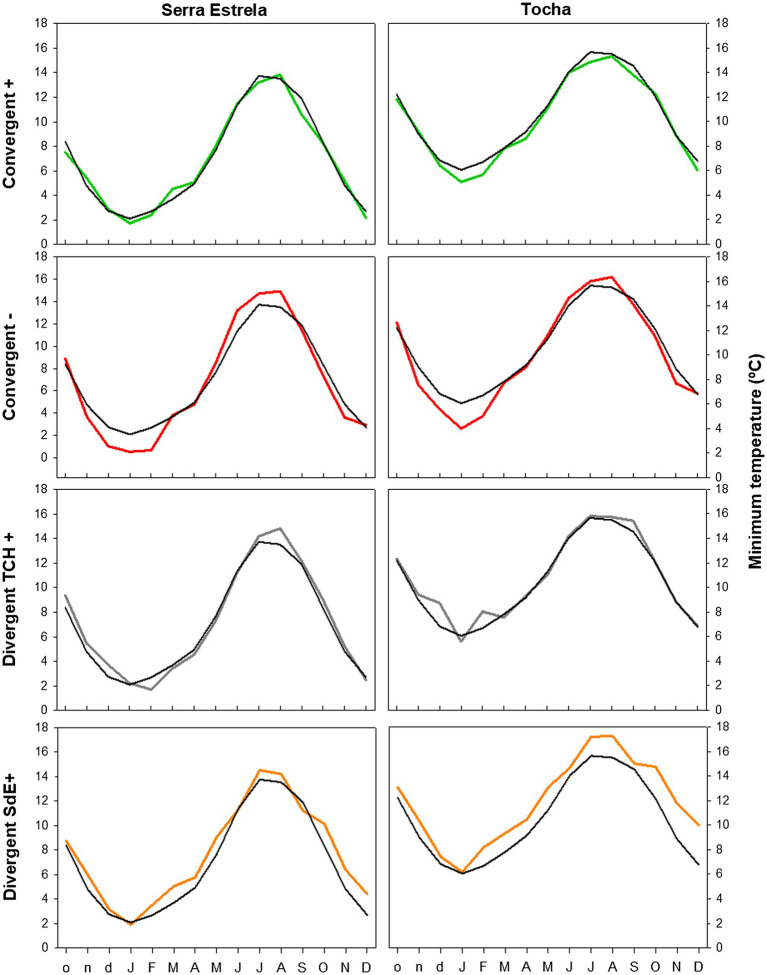
Monthly minimum temperature in SdE and TCH in the positive event years (convergent +; green line), negative event years (convergent −; red line), divergent years with TCH presenting a growth increment and SdE a growth reduction (Divergent TCH+; gray line), and the opposite (Divergent SdE+; orange line). Black line represents the average monthly minimum temperature for the period 1968–2008.

**Figure 6:**
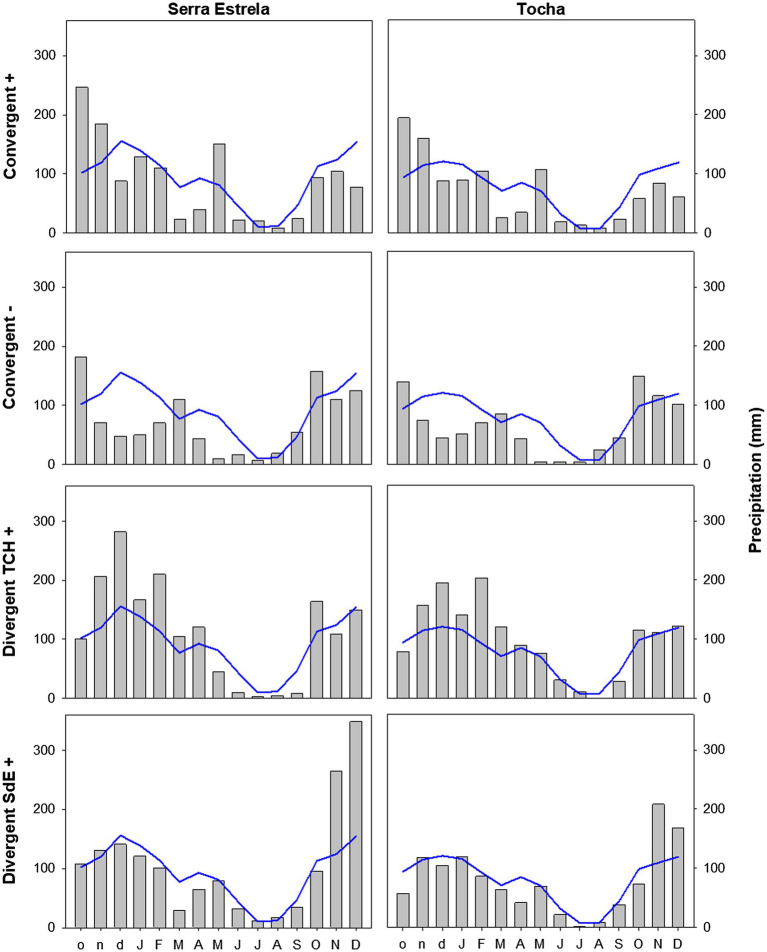
Monthly precipitation in SdE and TCH for the positive event years (Convergent +), negative event years (Convergent −), divergent years with TCH presenting a growth increment and SdE a growth reduction (Divergent TCH+), and the opposite (Divergent SdE+) represented by gray bars. Blue line represents the average monthly precipitation for the period 1968–2008.

**Figure 7:**
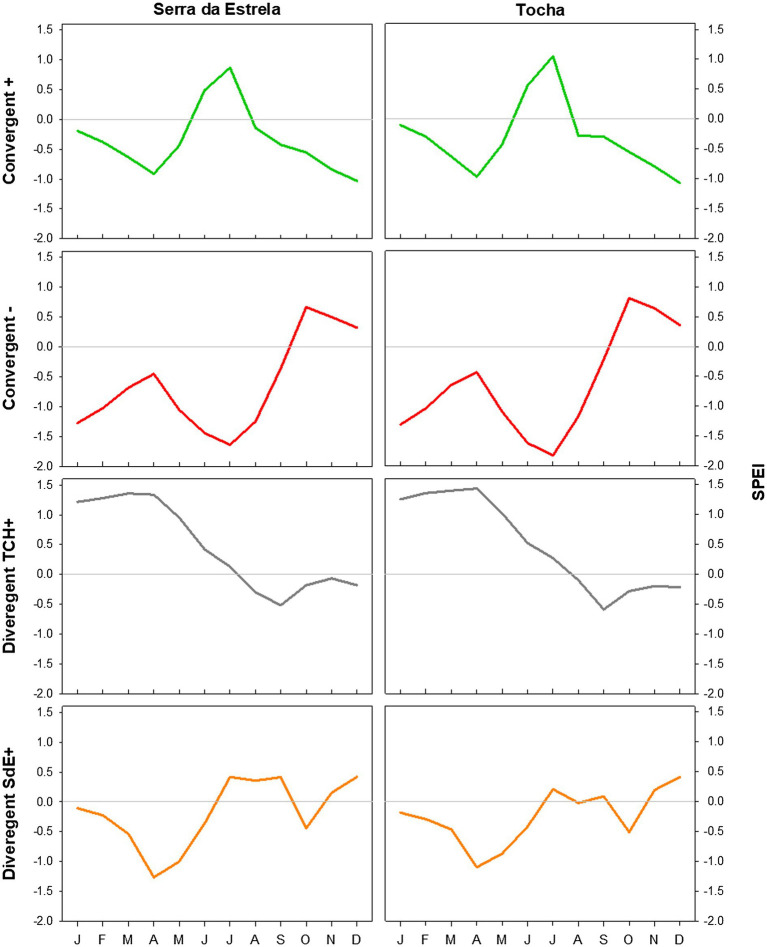
SPEI in SdE and TCH in the positive event years (convergent +; green line), negative event years (convergent −; red line), divergent years with TCH presenting a growth increment and SdE a growth reduction (Divergent TCH+; gray line), and the opposite (Divergent SdE+; orange line).

The analysis of minimum temperature, precipitation, and SPEI of event years revealed differences between those years and the average (Figures 5–7). The convergent positive event years were characterized by a minimum temperature identical to the long-term average in SdE and by below average minimum temperature in January, February, and in the summer months in TCH (0.75, 1.01, and 0.44°C below average, respectively; Figure 5). The precipitation pattern was similar between sites with precipitation below average in March and April (68 and 52% reduction in SdE, and 61 and 55% reduction in TCH), and above average in May (90% increment in SdE and 61% in TCH, Figure 6). SPEI was negative, corresponding to drought periods, from January to May and from August to December in both sites (Figure 7).

The convergent negative event years were characterized by below average minimum temperature in the previous winter (November, December, and January, 1.44°C below average in SdE and 1.55°C in TCH) and by above average minimum temperature from May to August, in both sites (1.16°C in SdE and 0.43°C in TCH; Figure 5). Precipitation was below average from previous November to July except for March (54% reduction in SdE and 57% in TCH; Figure 6). SPEI was negative from January to September in both sites (Figure 7).

Regarding the divergent event years, the analysis of the climatic parameters revealed that in years with growth increment in TCH and growth reduction in SdE (Divergent TCH+), precipitation was above average from previous November to May and in October in both sites (an increment of 80% in SdE and 50% in TCH; Figure 6), temperature was above average in August and September in SdE (0.7°C above average) and below average in TCH (0.64°C below average; Figure 5). SPEI was negative from July to December (Figure 7).

The years with growth increment in SdE and growth reduction in TCH (Divergent SdE+), temperature was 0.77°C above average in both sites throughout the year (Figure 5). In SdE, spring temperature was 1.14°C above average and in TCH 0.96°C. Precipitation was below average in February, April, and October in both sites, with a reduction of 14, 60, and 40% in SdE and 11, 46, and 30% in TCH, respectively (Figure 6). SPEI was negative from January to July and in October in both sites, and positive from July to September, however higher in SdE than TCH (Figure 7).

## Discussion

Extreme growth increment in *P. pinaster* was compared in two sites with different altitudes along an oceanic-continental gradient using a novel approach that divided event years in convergent and divergent. The results supported our initial hypotheses that convergent growth corresponds to years with above average precipitation in previous winter, and negative convergent growth to below average precipitation during the growing season. The analysis of the divergent growth years revealed that TCH is more dependent on precipitation and that SdE growth can be enhanced by warmer early spring temperatures. Convergence in tree growth represented a regional signal, whereas a local signal was detected by divergent growth reactions.

### Convergent Growth

Convergence growth corresponded to climatic responses previously observed in the Mediterranean region. Convergent growth increment was observed in years with above average previous winter and May precipitation, whereas growth reduction (convergent negative) was observed in years with below average spring precipitation and above average summer temperature. Positive event years were associated with precipitation in previous winter and spring, as previously reported for *P. pinaster* growing in Portugal (Vieira et al., 2009, 2017a; Campelo et al., 2013, 2015), Spain (Arzac et al., 2018; Caminero et al., 2018), and Italy (Mazza et al., 2015). The correlation analysis confirmed the positive signal with previous autumn and winter precipitation in tree-ring width. The importance of previous autumn and winter precipitation for tree growth could be related to the recharge of soil water reserves before the growing season (Pasho et al., 2012), which is critical for Mediterranean conifer species growing in drought-prone areas with long summer and shallow or rocky soils (Camarero et al., 2013). The analysis of SPEI supported the importance of precipitation for tree growth since these years were characterized by a positive SPEI from May to July, indicative of a wetness period (Vicente-Serrano et al., 2010). Increased water availability from May to July could support higher rates of cell production and thus induce the formation of wider tree rings. The maximum rate of cell production in *P. pinaster* trees growing in TCH Portugal was observed in March. After this period, the rate of cell production decreased, especially in trees under rain exclusion (Vieira et al., 2020).

As expected, negative event years were formed in dry years, with below average precipitation in previous winter and spring, above average summer temperatures, and a negative SPEI from previous winter to current summer. The identified negative convergent event years correspond to extreme drought years and were also detected in other studies analyzing extreme growth reduction in the Iberian Peninsula (Gazol et al., 2018; Sánchez-Salguero et al., 2018). Low water availability limits tree growth directly by reducing the rate of cambial cell division (Vieira et al., 2017b, 2019, 2020) and indirectly by reducing photosynthesis and the soluble sugars available for secondary growth (Cartenì et al., 2018). Low water availability associated with warmer temperatures can trigger stomatal closure (Roman et al., 2015) in isohydric species such as *P. pinaster* (Ripullone et al., 2007). Stomata close in response to declining water availability and rising atmospheric vapor pressure deficit to reduce water losses, and to prevent xylem cavitation and hydraulic failure (Roman et al., 2015; Garcia-Forner et al., 2016). By closing the stomata, the photosynthetic rate declines due to limited carbon uptake, which decreases the carbohydrates available for secondary growth (Macallister et al., 2019). Since secondary growth is a low priority sink in carbohydrate allocation (Heinrich et al., 2015), less carbon will be available for growth, and thus, the tree rings formed are narrower. The formation of very narrow tree rings in very dry and warm years is not exclusive of the Mediterranean region and has been reported in other environments, such as Central Europe lowlands (Neuwirth et al., 2007), France (Lebourgeois et al., 2010), and the Swiss Alps (Neuwirth et al., 2004).

### Divergent Growth

Divergent growth highlighted site differences and revealed a temperature signal in the high-altitude site. The years identified with growth increment in TCH and growth reduction in SdE (TCH+) were associated with above average precipitation. On the other hand, SdE+ years were characterized by below average spring precipitation and above average minimum temperatures. The precipitation pattern is identical in both sites, and the correlation analyses revealed that June precipitation was equally important for tree growth in both studied sites. However, regarding extreme divergent growth increments, TCH presented extreme growth increment in years characterized by above average precipitation, whereas in SdE extreme growth increment was observed in years with average precipitation but above average early spring temperature. This difference in precipitation dependency is also clear in SPEI. In TCH+ years SPEI is positive from January to July, whereas in SdE+ years is positive from July to September, indicating that TCH trees are more dependent on precipitation than SdE trees. The different sensitivities to precipitation and temperature observed between sites in divergent years revealed differences in site conditions. At high elevation sites, such as SdE, a warmer early spring can trigger an earlier start of cambial activity (Rossi et al., 2008; Moser et al., 2010), and thus a longer period of wood formation, which can result in the formation of wider tree rings (Vieira et al., 2020).

The above average October precipitation observed in TCH+ years and in positive convergent years could be related to the capacity of *P. pinaster* to resume secondary growth after the summer drought (Vieira et al., 2015). In fact, the only group of years that did not show above average precipitation in October were the years in which TCH showed growth reduction and SdE growth increment (SdE+). The resumption of cambial activity and wood formation in autumn results in the formation of an intra-annual density fluctuation (Campelo et al., 2007; Vieira et al., 2015; Battipaglia et al., 2016), which is common in Mediterranean species, and has been associated with the bimodal growth pattern (Camarero et al., 2010; Campelo et al., 2018). The extra growth band produced after the summer has been previously observed in TCH (Campelo et al., 2013, 2015; Vieira et al., 2015) and helps to explain the formation of wider tree rings in years with high precipitation in autumn.

### Mediterranean Forests Under Climate Change

In the near future, the Mediterranean Basin is predicted to be the most vulnerable region in Europe to climate change (Jacob et al., 2014). Climatic projections from the EURO-CODEX initiative using the Representative Concentration Pathways 4.5 (Jacob et al., 2014) predict an increase in the mean annual temperature from 3.3 to 4.1°C and a decrease of 11–17% of the total annual precipitation for the Mediterranean region. In SdE, the predicted changes are already in place, the average annual temperature increased nearly 1.5°C since 1950, and the total annual precipitation also decreased significantly (Figure 2). Although SdE trees presented a positive response to early spring temperatures, forming wider tree rings in those years, the negative correlation with summer temperatures and positive correlation with SPEI in summer months also indicates that tree growth is negatively affected by summer drought. Growth of SdE trees will be enhanced by warmer early spring temperatures whenever water shortage does not override the temperature effect. If temperature continues to increase and precipitation decreases, the productivity of SdE trees is expected to decrease. In TCH, the trend of temperature increase and precipitation decrease is not as marked as in SdE probably due to the proximity to the Atlantic Ocean, which can act as a buffer minimizing temperature variation.

Changes in temperature and precipitation regimes may increase drought risk, which can negatively affect trees’ physiological performance (Garcia-Forner et al., 2019), carbon allocation (Cartenì et al., 2018), and growth (Vieira et al., 2019). In fact, changes in intensity, duration, and frequency of droughts are responsible for many observed shifts on vegetation and forest dieback (DeSoto et al., 2020). Although we used a small dataset, with only three event years from each category, climatic conditions differ between event year categories, supporting that local adaptation will become more important under future climate change scenarios. The importance of local adaptation for species distribution under climate change has been demonstrated by Benito-Garzón et al. (2011) that modeled the potential distribution of *P. sylvestris* and *P. pinaster* in the Iberian Peninsula under climate change. They demonstrated the importance of including plasticity and genetic diversity among populations when predicting species distribution, mechanisms that can buffer or exacerbate processes leading to extinction risk.

## Conclusion

The new method used in this study, which consisted in dividing event years in convergent and divergent, revealed that convergent growth, whether positive or negative, was triggered by identical climatic conditions in both sites. In fact, the negative convergent years identified in our study corresponded to dry years identified across the Iberian Peninsula. Convergent event years revealed a regional climatic signal that represents the main climatic drivers in a broader geographic scale. Divergent event years, however, revealed climatic conditions at a local scale. Divergent growth was partly explained by temperature, revealing that *P. pinaster* trees from a high elevation took advantage of warmer early springs, probably due to an earlier start of the growing season, while *P. pinaster* from lowlands was negatively affected by it, probably due to increasing drought. Given the climatic projections for the Mediterranean region, divergent growth is expected to increase in the future, particularly between sites at low altitude near the coast and inland sites at high altitudes, suggesting that local adaptation will become more important. This new method revealed very interesting results, and its use in larger data sets will certainly help explain tree growth under climate change. The information gathered in this study gives valuable insights on the response of *P. pinaster* to extreme climatic events, allowing for more adjusted management strategies of Mediterranean pine forests.

## Data Availability Statement

The raw data supporting the conclusions of this article will be made available by the authors upon request to the corresponding author.

## Author Contributions

JV and FC designed the study, proposed the hypothesis tested, explored and analyzed the data, prepared figures and tables, and wrote the first draft of the manuscript. All authors contributed to the article and approved the submitted version.

### Conflict of Interest

The authors declare that the research was conducted in the absence of any commercial or financial relationships that could be construed as a potential conflict of interest. The handling editor declared a past collaboration with one of the authors FC.

## Footnotes

^1^ http://climexp.knmi.nl/start.cgi
